# Cloning and Heterologous Expression of the Grecocycline Biosynthetic Gene Cluster

**DOI:** 10.1371/journal.pone.0158682

**Published:** 2016-07-13

**Authors:** Oksana Bilyk, Olga N. Sekurova, Sergey B. Zotchev, Andriy Luzhetskyy

**Affiliations:** 1 Helmholtz-Institute for Pharmaceutical Research Saarland, Saarland University Campus, Building C2.3, 66123 Saarbrücken, Germany; 2 Department of Pharmacognosy, University of Vienna, 1090 Vienna, Austria; 3 Universität des Saarlandes, Pharmazeutische Biotechnologie, Building C2.3, 66123 Saarbrücken, Germany; University Paris South, FRANCE

## Abstract

Transformation-associated recombination (TAR) in yeast is a rapid and inexpensive method for cloning and assembly of large DNA fragments, which relies on natural homologous recombination. Two vectors, based on p15a and F-factor replicons that can be maintained in yeast, *E*. *coli* and streptomycetes have been constructed. These vectors have been successfully employed for assembly of the grecocycline biosynthetic gene cluster from *Streptomyces* sp. Acta 1362. Fragments of the cluster were obtained by PCR and transformed together with the “capture” vector into the yeast cells, yielding a construct carrying the entire gene cluster. The obtained construct was heterologously expressed in *S*. *albus* J1074, yielding several grecocycline congeners. Grecocyclines have unique structural moieties such as a dissacharide side chain, an additional amino sugar at the C-5 position and a thiol group. Enzymes from this pathway may be used for the derivatization of known active angucyclines in order to improve their desired biological properties.

## Introduction

Actinomycete bacteria represent a rich source of secondary metabolites with diverse chemical scaffolds and interesting biological activities. 43% of biologically active compounds of microbial origin were isolated from actinomycetes, most of them from representatives of the genus *Streptomyces* known to be prolific secondary metabolite producers [1]. One of the approaches to exploit biosynthetic potential of actinomycetes involves the expression of orphan secondary metabolite gene clusters in heterologous host. Therefore, construction of vectors capable of carrying entire biosynthetic gene clusters is of high interest.

Nowadays, cosmid vectors are routinely used for genomic libraries constructions, and they can accommodate between 31 kb and 44 kb of foreign DNA [2]. However, gene clusters encoding secondary metabolites may reach over 100 kb in size, and cosmid vectors simply do not have a capacity to stably maintain such large DNA segments. There exist several alternative replicons capable of accommodating larger DNA insertions: p15a, RK2 and F1 (used for construction of BACs). p15a is a low copy (about 15 copies per chromosome) replicon from *Escherichia coli* [3]. Its utility in maintaining large DNA fragments was demonstrated in the assembly of a 62.4 kb-large epothilone biosynthetic gene cluster [4]. RK2 replicons belong to the IncP incompatibility group and have been used for metagenomics library constructions [5]. Vectors constructed with RK2 replicon function in numerous Gram-negative bacterial species and have been transferred to Gram-positive bacteria, yeast and mammalian cells [6, 7]. Bacterial artificial chromosome (BACs) and P1 artificial chromosomes (PACs)-derived libraries are good alternatives to cosmid libraries when cloning of large DNA fragments is needed. PACs that combine features of BACs and bacteriophage P1 vectors can carry inserts ranging in size from 60 kb to 150 kb, whereas BACs are capable of accommodating and propagating of DNA fragments up to 700 kb (with an average insert size 150 kb). However, construction of BAC-derived libraries requires special equipment, is time-consuming, and expensive.

During the past years transformation-associated recombination (TAR) in yeast was successfully applied to assemble and clone large DNA fragments, including those harboring secondary metabolite gene clusters [8–11]. Originally, TAR method was developed for cloning of large genomic fragments without constructing and screening of genomic libraries [12]. This approach relies on homologous recombination between DNA of interest and short (~ 60 bp) “capture arms” of the TAR-vector. Advantage of the method is elimination of *in vitro* enzymatic reactions such as restriction and ligation and reducing the amount of DNA handling. In addition, a recently published approach combining TAR-cloning and CRISPR/Cas9 system demonstrates a 15-fold recombination increase during TAR-assisted cloning of the human genes [13].

Herein, we report construction of specialized TAR-dedicated vectors, and successful assembly and heterologous expression of a 36-kb long grecocycline biosynthetic gene cluster (*gre*) using TAR method. Fragments of the gene cluster were obtained using PCR and transformed into *Saccharomyces cerevisiae* together with the “capture” vector, resulting in the construct carrying the entire gene cluster. The obtained construct was successfully expressed in *S*. *albus*, yielding several grecocycline congeners. Apparently, PCR-introduced mutations were responsible for the biosynthesis of these metabolites, suggesting that error-prone PCR combined with TAR assembly can be used for generation of new natural products’ analogues.

## Methods and Materials

### Strains, growth conditions and media

For standard purposes *S*. sp. Acta 1362 and *S*. *albus* J1074—an *S*. *albus* G1 (DSM 41398) derivative with the defective SalGI restriction modification system, wild types and mutant were grown on 2% manitol and 2% soy bean meal, pH 7.5, prepared as solid medium and tryptone soy broth (TS broth), prepared as liquid medium, at 28 0C. For maintenance of *S*. *albus* pGRE apramycin was added to a final concentration of 50 μg/ml. For production a liquid medium DNPM (4% dextrin, 0,75% soytone, 0,5% baking yeasts and 2,1% MOPS, pH 6.8) was used. DNA manipulations were carried out in Escherichia coli BG2005 and the non-methylating *E*. *coli* ET12567/pUZ8002 was used to drive conjugative transfer of non-methylated DNA to *Actinobacteria* as described previously [14]. *E*. *coli* strains were grown on Luria-Bertani agar or liquid medium containing appropriate antibiotic for selection.

### General genetic manipulation and standard PCR

Standard molecular biology procedures were performed as described previously [2]. Isolation of plasmid DNA from *E*. *coli* and DNA restriction/ligation were performed by following the protocols of the manufacturers of the kits, enzymes, and reagents, Qiagen, Promega, NEB and Thermo Fisher Scientific. PCR reactions were performed by using Phusion High-Fidelity DNA polymerase (Thermo Fisher Scientific) for complementation and expression experiments and DreamTaq polymerase (Thermo Fisher Scientific) to verify mutants. Primers were purchased from Eurofins MWG Operon. The oligonucleotide primers that were used are listed in S1 Table. The oligonucleotide primers used to sequence pGRE are listed in S2 Table.

### Construction of vectors for cluster assembly

pCLY9 vector was constructed using Gibson assembly method from PCR products generated in the following manner. DNA fragment encompassing pA15 replication origin was amplified with primers 15aSOKf and 15aSOKr using pACYC184 DNA template [15]. DNA fragment encompassing apramycin resistance gene, oriT, attP and phage VWB integrase gene was amplified using primers SOK15af and SOK15ar from pSOK804 DNA template. pCLY10 vector was constructed using Gibson assembly method from PCR products generated as follows. DNA fragment encompassing yeast centromere/replication origin CEN6/ARS4 and *LEU2* marker was amplified with primers CEN_LEUf and CEN_LEUr using pRS415 DNA template (SnapGene, USA). DNA fragment encompassing 15a replication origin, apramycin resistance gene, oriT, attP and phage VWB integrase gene was amplified using primers CL9Lf and CL9Lr from the pCLY9 DNA template. pCLY11 vector was constructed using Gibson assembly method from PCR products generated as follows. DNA fragment encompassing yeast centromere/replication origin CEN6/ARS4, *LEU2* marker, apramycin resistance gene, oriT, attP and phage VWB integrase gene was amplified with primers CLYbacf and CLYbacr using pCLY10 DNA template. DNA fragment encompassing BAC replication origin and associated partition genes was amplified using primers BACclyf and BACclyr from the pCC1BAC DNA template (Epicentre, USA). Sequences of the oligonucleotide primers mentioned above are presented in S1 Table.

### Gene complementation cassettes

To generate the expression vector pUWLgreM2, *greM2* was amplified by PCR from *S*. sp. Acta 13–62 chromosomal DNA. Suitable restriction sites (*Xba*I and *Kpn*I) were introduced upstream and downstream from the gene, using primers. Also forward primer was carrying 21p promoter. To obtain suitable shuttle vector, pUWLCre was treated with *Kpn*I/*Xba*I and 8kb-fragment was eluted from the agarose gel (done according to the manufacturer, Promega). 2.4 kb PCR-fragment was digested with *Xba*I and *Kpn*I and ligated into pUWL vector, to generate pUWLgreM2.

### *gre* gene cluster assembly using TAR

Homologous sequences to the *gre* cluster were introduced into pCLY10 via PCR. Primers were designed to overlap 38 bp of the 500 bp long region upstream from the *gre* cluster (forward primer) and 38 bp of the 130 bp long region downstream from the cluster (reverse primer). Prior to the PCR pCLY10 was treated with *Hind*III, obtained PCR-product was treated with *Dpn*I for 3 hours. *gre* cluster was split into 3 parts: region 1, region 2 and region 3 (R1, R2 and R3), each size in range from 10 to 14 kb. R1 and R3 were chosen for the first step of the cluster assembly. Primers for R1 were designed to overlap with 38bp pCLY10 (forward primer) and R3 (reverse primer); R3-primers had 38-bp long overlaps with R1 (forward primer) and pCLY10 (reverse primer). *S*. sp. Acta 13–62 chromosomal DNA was used as a template for PCR amplifications. Mixture of obtained PCR-products (R1, R3 and pCLY10) was transformed into *Sac*. *cerevisiae* BG4742 (LEU) cells in concentration 100 ng each. After 4 days of incubation at 30°C, transformants were picked from the plates, plasmid DNA was isolated and transformed into *E*. *coli* BG2005 cells. Verification of obtained constructs revealed pCLY10 carrying R1 and R3 regions (pCR1.R3). *Sca*I restriction site was introduced between R1 and R3 artificially using primers, thus giving possibility to introduce R2 into pCR1.R3. Due to the problems with PCR, R2 was split into two parts: R2.1 (5743 bp) and R2.2 (7586 bp), overlap between R2.1 and R1 was 110 bp, R2.2 and R3 49 bp, R2.1 and R2.2 41 bp. To amplify pCLY10, R1 and R3 MasterAmp^™^ Extra-Long PCR Kit from Epicentre was used, to amplify R2.1 and R2.3 polymerase from the kit was substituted with Phusion High-Fidelity DNA Polymerase from Thermo Fisher Scientific. Mixture of R2.1, R2.2 PCR-products and pCR1.R3, treated with *Sca*I, was transformed into yeast cells. Transformants were picked from the plates after 4 days incubation at 30°C, prior to plasmid DNA isolation, colony-PCR was done. Primers were specific to the DNA sequence that can be present only in a reassembled gene cluster construct. 22 positive clones were picked out of 95 after PCR, 4 clones were selected randomly and plasmid DNA was isolated. After verification by restriction mapping all four clones were positive for containing pR1.R3 reassembled with R2.1 and R2.2 (pGRE).

### Conditions for amplification of R1, R2.1, R2.2, R3 and pCLY10

To amplify pCLY10, R1, R2.1, R2.2 and R3 Master-Amp^™^ Extra-Long PCR Kit (Epicentre) was used: PreMix 4 for amplification R3 and R2.2, PreMix 8 for amplification R1 and R2.1. Phusion High—Fidelity DNA (Thermo Fisher Scientific) polymerase was used to amplify R2.1 and R2.2, and Master-Amp^™^ Extra-Long Polymerase Mix (Epicentre) for R1 and R3 amplification. PCR conditions for R1, R2 and R3 amplification are listed in S3 Table.

### Transformation of yeast

Yeast transformation was done as described before [16].

### Isolation of plasmid DNA from yeast

Plasmid DNA was isolated from the yeast cells using Wizard Plus SV Miniprep kit from Promega. 37,5 Units of Longlife Zymolyase (G-Biosciences, USA) were added to P1 buffer, samples were incubated at 37°C for 30–60 min. Further steps were done according to the manufacturer.

### Purification of compounds

*S*. *albus* pGRE was cultivated in DNPM medium in shaking flasks, total volume 5 L at 30°C, 200 rpm for 5 days. Mixture of culture broth and biomass was extracted with 5 L of ethylacetate (EtAc) and led to 145 mg of crude extract. The extract was purified by HPLC (HTec C18 column, 250 × 10 mm, 5 μm, A = H2O + 0.1% FA, B = ACN + 0.1% FA) with gradient starting from 5% B to 95% B in 28.2 min. Flow 5.8 ml/min, injection volume 40 μl of an MeOH extract (c = 5.3 mg/ml), detection wavelength λ = 420 nm.

### Production analysis

For production analysis 3 ml of 24-h old pre-culture of *S*. *albus* pGRE was inoculated into 50 ml of DNPM media and grown for 4 days at 30°C with agitation at 200 rpms. The culture broth was extracted with ethylacetate, samples were evaporated, dissolved in 500 μl of methanol and subjected to LC-MS analysis.

Standard measurements were performed on a Dionex Ultimate 3000 RSLC system using a BEH C18, 100 x 2.1 mm, 1.7 μm d_p_ column (Waters, Germany). Separation was achieved by a linear gradient from (A) H_2_O + 0.1% FA to (B) ACN + 0.1% FA at a flow rate of 600 μL/min at 45°C. The gradient was initiated by a 0.5 min isocratic step at 5% B, followed by an increase to 95% B in 18 min to end up with a 2 min step at 95% B before re-equilibration under the initial conditions. UV spectra were recorded by a DAD in the range from 200 to 600 nm. MS data was acquired with an Amazon Speed 3D ion trap mass spectrometer (Bruker Daltonics, Germany) using the Apollo ESI source. Mass spectra were acquired in centroid mode ranging from 150 to 1500 m/z.

High-resolution measurements were performed on a Dionex Ultimate 3000 RSLC system using a BEH C18, 100 x 2.1 mm, 1.7 μm d_p_ column (Waters, Germany). Separation of a 1 μl sample was achieved by a linear gradient from (A) H_2_O + 0.1% FA to (B) ACN + 0.1% FA at a flow rate of 600 μL/min and 45°C. The gradient was initiated by a 0.5 min isocratic step at 5% B, followed by an increase to 95% B in 18 min to end up with a 2 min step at 95% B before re-equilibration under the initial conditions. UV spectra were recorded by a DAD in the range from 200 to 600 nm. The LC flow was split to 75 μL/min before entering the maXis 4G hr-ToF mass spectrometer (Bruker Daltonics, Germany) using the Apollo ESI source. Mass spectra were acquired in centroid mode ranging from 150–2500 m/z at a 2 Hz scan rate.

## Results

### Grecocycline biosynthetic gene cluster identification and bioinformatic analysis

**Fig 1:**
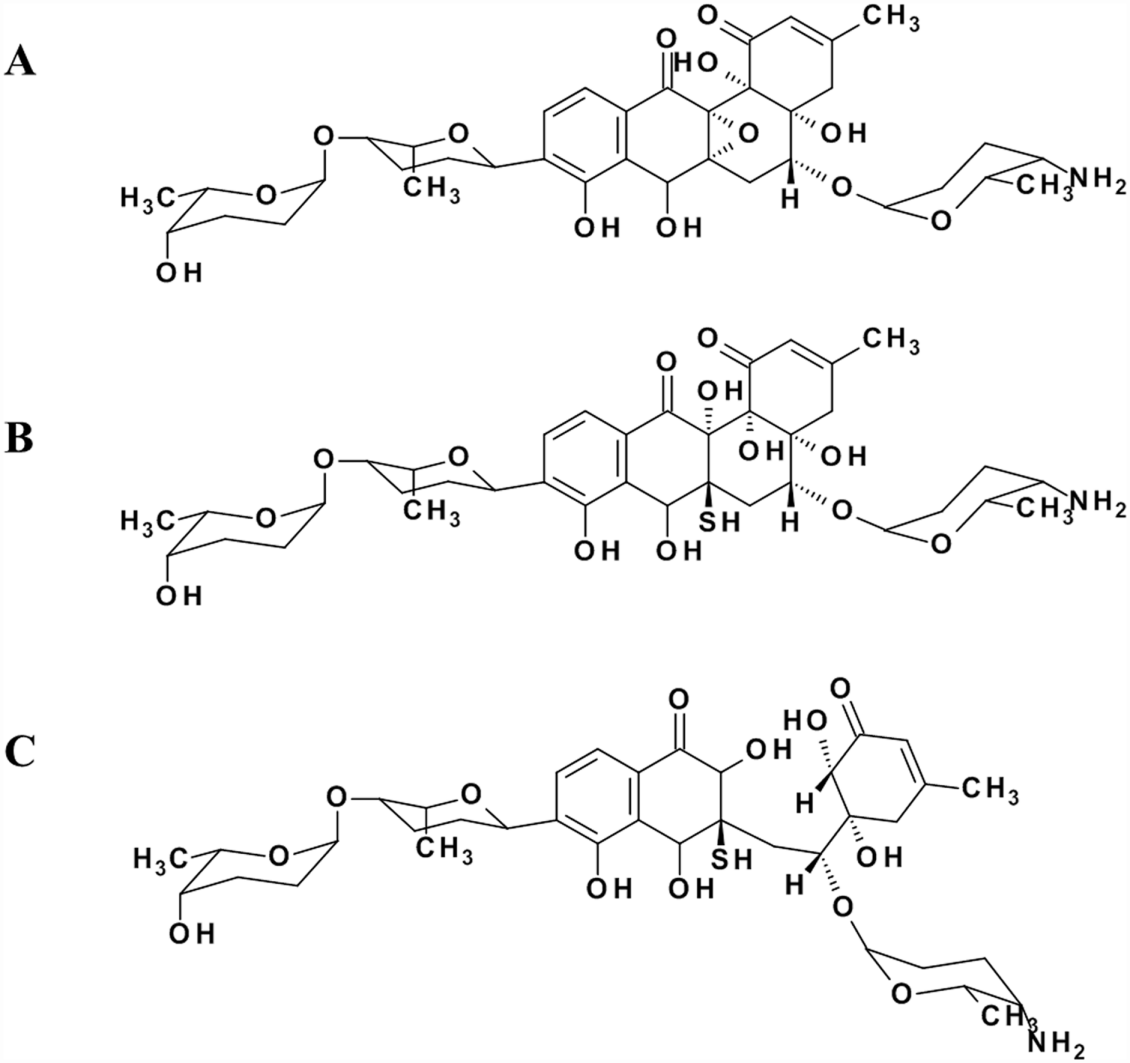
Structures of grecocylines A (A), B (B) and C (C).

**Fig 2:**
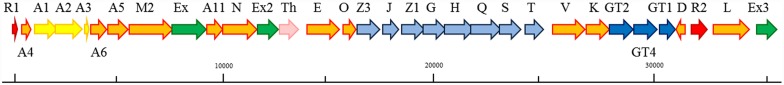
Grecocycline biosynthetic gene cluster (*gre*) from *S*. sp. Acta 13–62. In yellow are labeled genes encoding type II PKS, orange—genes involved in angucycline formation, blue—genes involved in biosynthesis of rhodinose and α-tolyposamine, dark blue—genes encoding glycosyltransferases, pink—gene encoding putative thioesterase, green—efflux, red—regulatory genes. Dashed arrow indicates formation of shunt product grecocycline C.

The *Streptomyces* sp. Acta1362 draft genome contains 8,710,318 base pairs with an average GC content of 71% and includes 8,026 open reading frames. Using antiSMASH [17] we have predicted 41 potential biosynthetic gene clusters, among them two encoding type II PKS systems. As grecocyclines contain sugar moieties [18] (Fig 1), the presence of genes encoding glycosyltransferases and deoxysugar biosynthesis enzymes are expected to be present within the biosynthetic gene cluster. Indeed, three genes for glycosyltransferases were identified in one of the type II PKS gene clusters, along with the genes required for deoxysugars biosynthesis, genes encoding post-PKS tailoring enzymes, regulatory and transporter genes. Altogether they comprise the putative grecocycline biosynthetic gene cluster (*gre* cluster) that encompassing 32 predicted open reading frames (ORFs) (Fig 2, Table 1).

**Table 1:** Deduced function of ORFs in the grecocycline biosynthetic gene cluster.

Polypeptide	aa	Similar protein	Acc. number	Identity	Proposed function
GreR1	216	PgaR1; *Streptomyces* sp. PGA64	AHW57766.1	68%	transcriptional regulator
GreA4	110	LndF; *Streptomyces globisporus*JadI; *Streptomyces venezuelae*	AAU04837.1AAO65345.1	82%81%	polyketide cyclase
GreA1	354	PgaA; *Streptomyces* sp. PGA64	AAK57525.1	85%	ketosynthase alfa
GreA2	405	PgaB; *Streptomyces* sp. PGA64	AAK57526.1	73%	ketosynthase beta
GreA3	92	PgaC; *Streptomyces* sp. PGA64	AAK57527.1	72%	acyl carrier protein
GreA6	262	PgaD; *Streptomyces* sp. PGA64	AAK57528.1	87%	ketoreductase
GreA5	315	UrdL; *Streptomyces fradiae*PgaL; *Streptomyces* sp. PGA64	AAF00205.1AAK57529.1	79%74%	aromatase
GreM2	794	PgaM; *Streptomyces* sp. PGA64	AAK57530.1	67%	two-domain mono-oxygenase
GreEx	503	PgaJ; *Streptomyces* sp. PGA64	AAK57531.1	53%	transporter
GreA11	236	OvmF; *Streptomyces antibioticus*	CAG14972.1	63%	4'-phosphopantetheinyl transferase
GreN	515	PgaI; *Streptomyces* sp. PGA64	AAK57534.1	84%	acyl-CoA carboxylase, beta-subunit
GreEx2	391	uncultured soil bacterium V167	ACX83629.1	59%	putative major facilitator transporter
GreTH	305	*Streptomyces roseoverticillatus*	WP_030366371.1	63%	thioesterase
GreE	498	PgaE; *Streptomyces* sp. PGA64	AAK57522.1	97%	monooxygenase
GreO	199	UrdO; *Streptomyces fradiae*	AAF00220.1	62%	reductase
GreZ3	341	PgaZ3; *Streptomyces* sp. PGA64	AHW57779.1	45%	TDP-hexose-4-ketoreductase
GreJ	384	PgaC1; *Streptomyces* sp. PGA64	AHW57776.1	80%	TDP-hexose-4-aminotransferase
GreZ1	200	PgaZ1; *Streptomyces* sp. PGA64	AHW57777.1	74%	TDP-hexose-3,5-epimerase
GreG	356	PgaG; *Streptomyces* sp. PGA64	AHW57786.1	75%	TDP-hexose synthetase
GreH	337	PgaH1; *Streptomyces* sp. PGA64	AHW57787.1	83%	TDP-hexose-4,6-dehydratase
GreQ	435	PgaQ; *Streptomyces* sp. PGA64	AHW57788.1	86%	TDP-hexose-3,4-dehydratase
GreS	465	PgaS; *Streptomyces* sp. PGA64	AHW57789.1	71%	TDP-hexose-2,3-dehydratase
GreT	328	PgaT; *Streptomyces* sp. PGA64	AHW57790.1	66%	TDP-hexose-3-ketoreductase
GreV	254	LanV; *Streptomyces cyanogenus*	AAD13552.1	62%	Reductase homolog
GreK	497	SaqE; *Micromonospora* sp. Tu 6368	ACP19351.1	68%	putative oxygenase
GreGT2	379	SaqGT5; *Micromonospora* sp. Tu 6368	ACP19370.1	59%	glycosyltransferase
GreGT4	425	Lcz36; *Streptomyces sanglieri*	ABX71153.1	54%	glycosyltransferase
GreGT1	392	SaqGT3; *Micromonospora* sp. Tu 6368	ACP19364.1	59%	glycosyltransferase
GreD	217	FrnE; *Streptomyces roseofulvus*	AAC18100.1	56%	DSBA oxidoreductase
GreR2	118	*Streptomyces* sp. CNH287	WP_027750658.1	59%	HxlR family transcriptional regulator
GreL	227	*Actinoplanes missouriensis*	WP_014442785.1	40%	putative monooxygenase
GreEx3	542	*Streptomyces* sp. W007	WP_007448654.1	95%	MFS transporter

Several genes involved in the angucycline formation and maturation are present in the cluster: 1) the type II PKS gene set is represented by *greA1*, *greA2* and *greA3* encoding alpha and beta ketosynthases (KSα and KSβ) and acyl carrier protein (ACP), respectively; 2) two cyclase encoding genes—*greA4* and *greA5*; 3) three ketoreductase encoding genes–*greA6*, *greV* and *greO*; 4) four oxygenase genes–*greM2*, *greE*, *greL* and *greD*; 5) one gene, *greA7*, encoding 4'-phosphopantetheinyl transferase presumably responsible for generation of holo-ACP; and 6) the gene encoding a decarboxylase, *greN*. Interestingly, *greD* is the only gene transcribed from the antisense strand. An additional gene encoding enzyme presumably acting on the angucyclic core of the grecocyclines is *greTh*. The deduced amino acid sequence of GreTh is highly similar to thioesterases and may be involved in formation of the thiol group in grecocycline B.

Two different deoxysugars are attached to the angucycline core: L-rhodinose and L-tolyposamine. Eleven of the thirty-two genes in the *gre* cluster encode enzymes that are presumably involved in the biosynthesis and transfer of sugars to the polyketide. The genes *greG*, *greH*, *greS*, *greT*, *greQ*, *greZ1* and *greZ3* encode enzymes that are predicted to be involved in the formation of NDP-rhodinose from D-glucose-1-phosphate. Additionally, *greJ* encodes NDP-hexose-4-aminotransferase that probably transfers amino group to the hexose to form L-tolyposamine.

**Fig 3:**
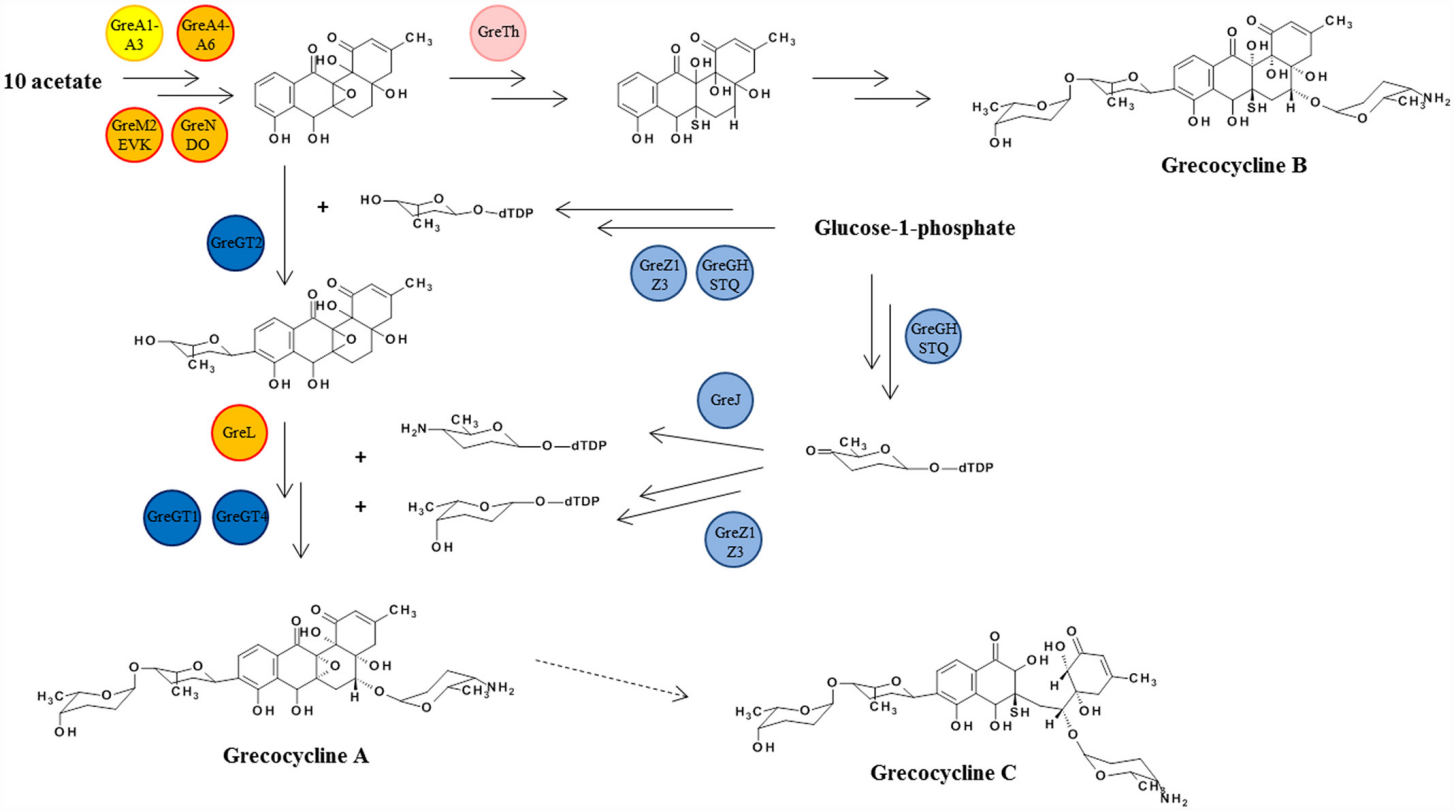
Proposed biosynthesis of grecocyclines. In circles are indicated enzymes putatively involved in particular biosynthetic steps.

Grecocyclines contain three sugar moieties, and a corresponding number of glycosyltransferase encoding genes was identified in the *gre* cluster—GreGT1, GreGT2 and GreGT4. *gre*GT2 encodes a protein that is homologous to SaqGT5 and UrdGT2 [19, 20] and may be involved in the attachment of L-rhodinose at the C-9 position of the aglycon, similar to urdamycins and saquayamycins. Probably, GreGT1 is responsible for the introduction of a second L-rhodinose unit, as it is highly similar to SaqGT3, SaqGT4, LanGT1 which are responsible for the extension of an oligosaccharide chain in angucyclines [19–21]. To our knowledge, grecocyclines are the first angucyclines that carry the sugar moiety at C-5 position of the angucyclic core. Furthermore, α-tolyposamine is a very rare deoxysugar in natural products, so far found only in compound BU-4514N from *Microtetraspora* sp. [22]. It seems plausible that GreGT4 is transferring this aminosugar to the C-5 position of grecocyclines. The deduced amino acid sequence of GreGT4 shares 54% identity with Lcz3 from the lactanomycin gene cluster and 50% identity with UrdGT1a involved in urdamycin biosynthesis[23, 24]. As mentioned above, *greL* encodes monooxygenase that does not have a predicted function, and might be a potential candidate for hydroxylation of the aglycon at C-5 prior to the sugar attachment (Fig 3).

Two regulatory (*greR1*, *greR2)* and three transporter genes (*greEx*, *greEx2* and *greEx3)* were identified in the *gre* cluster. *greR1* is located at the 5’ end of the gene cluster, and encodes a protein with the similarity to transcriptional regulators of the OmpR family [25]. Deduced amino acid sequence of GreR1 is very similar to JadR1 and LndI. Disruption of *lndI* caused complete loss of landomycin E production in *S*. *globisporus*, while its overexpression led to an increase in the antibiotic production [26, 27]. LndI is an autoregulator as it can bind to its own promoter region as well as to the promoters of the *lnd* structural genes [28]. Presumably, GreR1 plays the same role in the grecocycline biosynthesis. GreR2 is homologous to the HxlR family of transcriptional regulators. GreEx, GreEx2 and GreEx3 are similar to MFS transporters and probably are exporting grecocyclines out of the cell.

All the attempts to generate the deletion mutants in *S*. sp. Acta 13–62 in order to confirm the identity of the *gre* cluster failed, and therefore we decided to assemble the gene cluster for heterologous expression.

### Construction of the TAR assembly vectors

**Fig 4:**
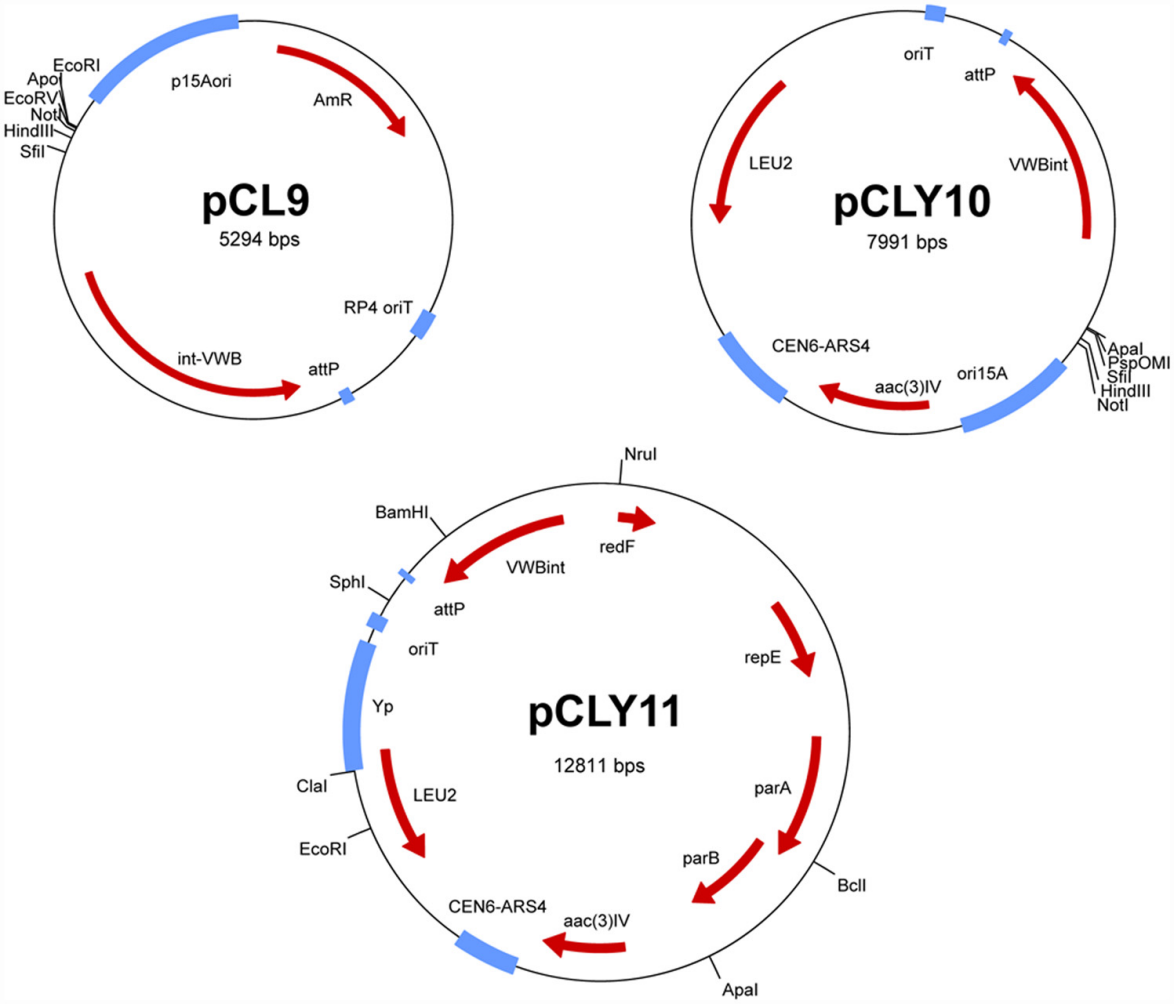
Physical maps of the vectors constructed in this study (see text for details).

To construct vectors capable of harboring large DNA fragments, a bifunctional plasmid pCLY9 (Fig 4) representing a hybrid between pACYC184 [29] and pSOK804 [30] was assembled using Gibson ligation method [31] (see Materials and Methods for details). In particular, ColE1 replicon in pSOK804 was replaced with that of p15A, yielding a conjugative shuttle vector pCLY9 that could be transferred to *Streptomyces* via intergeneric conjugation and site-specifically integrated into the chromosome. Next, pCLY9 was used in an attempt to assemble several gene clusters ranging in size from 30 kb to 50 kb using Gibson ligation method (data not shown). However, none of these attempts were successful, most likely due to formation of secondary structures by single stranded GC-rich DNA generated during exonuclease “chew-back” which would prevent proper alignment of complimentary strands during assembly.

To circumvent this problem, two vectors carrying elements allowing propagation in yeast were constructed. pCLY10 (Fig 4) was generated via insertion of yeast LEU2 marker gene and CEN-ARS4 centromere/replication origin elements from plasmid pRS415 [32] into pCLY9 via Gibson assembly (see Materials and Methods). pCLY11 was derived from pCLY10 by replacing the p15a replication origin with part of the pCC1BAC vector harboring F-factor replicon and having a copy control function. Both pCLY10 and pCLY11 were shown to propagate stably in *E*. *coli* and yeast *Sac*. *cerevisiae*, and to be efficiently transferred to *S*. *noursei*, *S*. *venezuelae and S*. *albus*. pCLY10 was chosen for grecocyline biosynthetic gene cluster assembly.

### Grecocylcine gene cluster assembly in yeast

**Fig 5:**
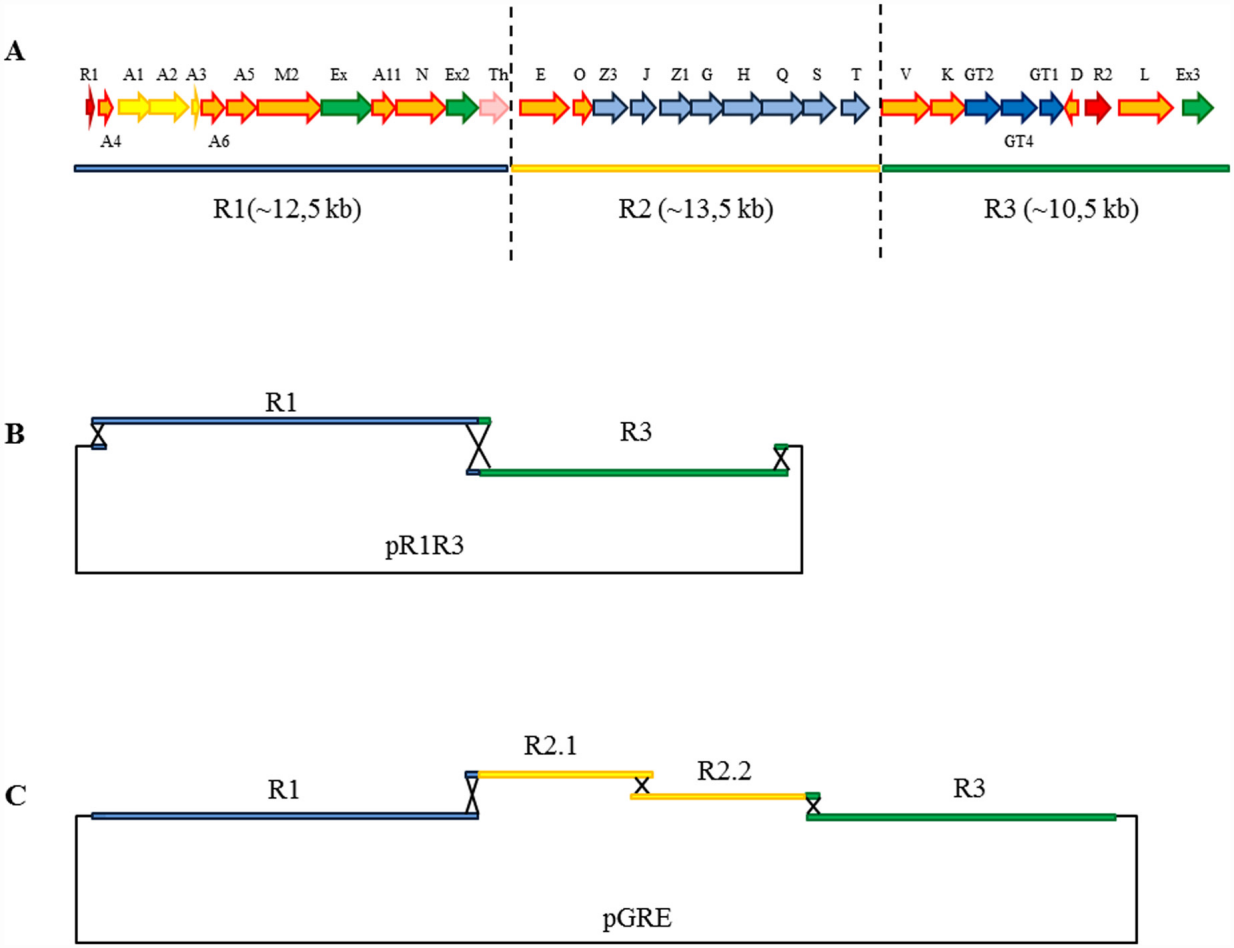
Strategy for assembling the grecocycline biosynthetic gene cluster using TAR. A–*gre* cluster divided on R1, R2 and R3; B—assembly of pR1.R3; C—assembly of pGRE.

To express the gene cluster in the heterologous host, we targeted appropriate DNA region for TAR cloning (Fig 5). Previous reports showed that short 40- to 70-bp DNA-specific targeting sequences were enough for successful TAR cloning [33]. The grecocycline biosynthetic gene cluster was split into three regions, R1, R2 (R2.1 and R2.2) and R3, overlapping with each other and with the shuttle vector (pCLY10) by 38 bp. The capture arms to the *gre* cluster were introduced into pCLY10 via PCR to generate pCLY10ol. Primers were designed to overlap 38 bp of the 500 bp long region upstream from the *gre* cluster (forward primer) and 38 bp of the 130 bp long region downstream from the cluster (reverse primer). Assembly of the *gre* cluster was done in two steps: 1) capture of R1 and R3 on the shuttle vector to give pR1R3; 2) capture of R2 on pR1R3 to yield pGRE. R1, R2 and R3 were amplified in PCR reaction with the primers carrying homologous arms to the adjacent DNA regions. After transformation of *Sac*. *cerevisiae* BY4742 cells with the mixture of obtained PCR-products (R1, R3 and pCLY10ol), positive clones were identified by PCR and confirmed by restriction mapping to yield pR1R3 construct. Next, linearized pR1R3 and R2 (R2.1 + R2.2) were transformed into yeast cells, yielding an assembly of pGRE—a construct carrying the entire *gre* biosynthetic gene cluster. On this stage the efficiency of TAR was approximately 23% (see Methods and Materials section for further information). The 44-kb pGRE construct was stably maintained in *E*. *coli*.

### Heterologous expression of the assembled *gre* gene cluster

**Fig 6:**
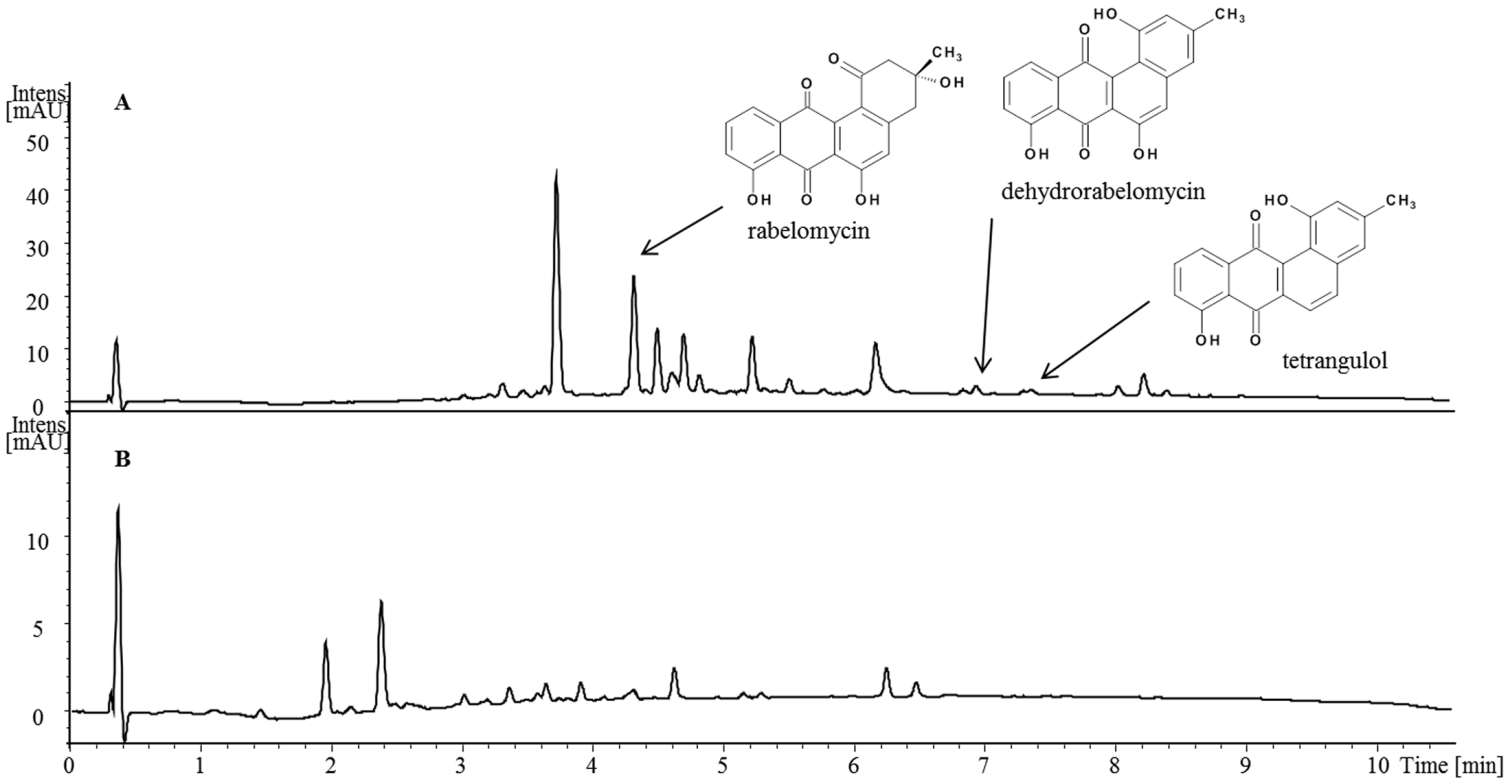
Chromatogramof the HPLC-ESI/MS analysis of crude extract from *S*. *albus* pGRE (A) and *S*. *albus* J1074 (B). Arrows indicate ions corresponding to rabelomycin, dehydrorabelomycin and tetrangulol. Wavelength 420 nm.

For the heterologous production, we chose S. *albus* J1074, a model streptomycete commonly used as a host for heterologous expression of various biosynthetic gene clusters, and for which genome sequence has recently become available [34]. pGRE was introduced into the genome of *S*. *albus* J1074 by conjugative transfer from *E*. *coli*, and the resulting recombinant strain was analyzed for production of grecocyclines (see Materials and Methods). *S*. *albus* strain carrying pGRE (*S*. *albus* pGRE) was found to produce a series of angucyclines, as revealed by the ESI-MS analysis (Fig 6, S1 and S2 Figs).

To determine the chemical structures of produced compounds, *S*. *albus* pGRE was cultivated in 5 L-scale to yield ~130 mg of crude extract solids, from which ten fractions were purified and subsequently analyzed by LC-MS. Three compounds from fractions N4, N9 and N10 were assigned to known angucycline intermediates or shunt products, based on the UV/Vis spectra and the accurate mass. The compound from fraction N4 was identified as rabelomycin (m/z = 338.07863), fractions N9 and N10 were shown to contain dehydrorabelomycin (m/z = 320.06741) and tetrangulol (m/z = 304.07257), respectively. No glycosylated products have been detected after the heterologous expression of assembled *gre* cluster in *S*. *albus*. Although no grecocyclines were detected in the crude extracts via LC-MS analysis, it was obvious that the cloned biosynthetic gene cluster is responsible for grecocycline precursor’s biosynthesis, since several known angucyclines were observed. To identify the reason why after the heterologous expression of cloned *gre* gene cluster no grecocyclines were produced we have sequenced the pGRE plasmid using primer walking, covering approximately 97,1% of the construct. Analysis of the sequenced data revealed twenty six nucleotide substitutions, presumably introduced during PCR. All the identified mutations are listed in Table 2 and discussed in the Discussion section.

**Table 2:** List of mutations introduced into the *gre* genes during amplification*.

Protein	Mutation	Proposed function
GreA5	A217T	aromatase
GreM	A2T; R339C; G407C; P665L; R734C; E747G	bifunctional oxygynase-reductase
GreN	I353V	carboxylase
GreK	V79V**; K101K**; L387P;	putrative oxygenase
GreGT4	W22R	glycosyltransferase
GreGT1	A291A**	glycosyltransferase
GreD	E147V	DSBA oxidoreductase
GreL	P104T; R106Q	putative monooxygenase
GreEx3	I322F; A500V	MFS transporter

*—mutations in the intergenic regions are not included

**—nucleotide substitution do not influence amino acid sequence

Eight nucleotide substitutions were identified in the DNA region encoding GreM2. Inactivation of *greM2* homologs in urdamycin and landomycin biosynthetic pathways led to accumulation of rabelomycin, tetrangulol and other shunt products [35, 36], preventing subsequent conversion of aglycon into the glycosylated natural product.

To complement mutated region encoding *greM2*, a native *greM2* was placed under the control of the strong synthetic 21p promoter and cloned into pUWL vector. Introduction of this construct into the S. *albus* pGRE strain did not change the spectrum of the compounds observed after the expression of assembled *gre* cluster alone. This result may reflect an unbalanced expression of *greM2* and the rest of the *gre* cluster from different plasmids.

## Discussion

For many decades actinomycetes have been the source of numerous pharmaceutically useful compounds which were developed into antibiotics, immunosuppressive, antiproliferative and antiparasitic drugs [37]. The fast progress in DNA sequencing technologies applied to genome sequencing revealed the wealth of natural product biosynthetic gene clusters in the genomes of actinomycetes, most of which remain cryptic [38]. One approach to access chemical diversity encoded by the cryptic/silent biosynthetic gene clusters is heterologous expression, while cloning of large gene clusters still represent a challenge. Therefore, technics for large DNA fragments cloning are of great interest.

In this study, we used TAR method to assemble and heterologously express grecocycline biosynthetic gene cluster. The entire 36-kb gene cluster was successfully captured on the shuttle vector carrying short (38-bp) homologous shoulders. We accomplished cluster assembly in two steps. The advantage of step-by-step strategy is very fast and effortless acquisition of all parts in large quantities, which facilitates their joining during TAR-assisted assembly. The only limiting step may be finding of the appropriate conditions for DNA amplification via PCR. The composition of transformation mixture used in TAR is thus free from excessive and non-related DNA generated when a digested genomic DNA mixture is used. The latter strategy may significantly reduce the recombination frequency and make assembly problematic. Hence, TAR-assisted cloning with only essential fragments may increase frequency of the correct assembly in a fold range. Another advantage of the PCR-based TAR assembly is introduction of homologous shoulders directly at the ends of the targeted DNA sequence. As was shown before, homologous recombination is much more efficient between TAR vector and the DNA sequence located closer to the ends compared to internally imbedded [39]. Application of a CRISPR/Cas9 system to introduce double-strand breaks close to the ends of the desired genomic fragment resulted in a dramatic increase in the fraction of gene-positive colonies [13]. The numbers of positive yeast clones from this study (23%) are comparable with efficiency of CRISPR/Cas9-mediated TAR cloning (32%). However, the disadvantage of the PCR-based part generation is certainly high risk of mutations, especially when long fragments are synthesized by DNA polymerases from GC-rich DNA. Therefore, the result of a seemingly correct assembly (e.g. verified only via restriction endonuclease digestion patterns) is to a large extent unpredictable, since absence of expected product can be either due to mutations introduced by PCR, or other reasons. On the other hand, randomly introduced mutations may provide an advantage in terms of generation of new analogues. Such method can be applied to any cloned gene cluster for which heterologous expression have been verified. Overlapping parts of such cluster can be amplified by error-prone PCR, re-assembled using TAR, and heterologous hosts carrying mutagenized clusters screened for new analogues or enhanced production of target compound.

The potential usefulness of this approach is in part supported by the expression of re-assembled *gre* gene cluster in *S*. *albus*. Undoubtedly, the angucycline compounds, which we have isolated, are produced after the expression of the *gre* biosynthetic gene cluster from pGRE, since the host *S*. *albus* lacks type II PKS-encoding genes. Although no grecocycline have been detected after heterologous expression of the *gre* gene cluster, the recombinant strain produced rabelomycin, dehydrorabelomycin and tetrangulol, known intermediates and shunt products formed during biosynthesis of angucycline polyketides [36, 40–42]. These data are also in agreement with the mutations detected in the *greM2* gene on pGRE. The deduced amino acid sequence of GreM2 shows significant similarity to the bifunctional oxygenase-reductases from the landomycin (LndM2, LanM2), gaudimycin (PgaM), and jadomycin (JadF, partially) biosynthetic pathways. LanM2 and PgaM belong to the family of short-chain alcohol dehydrogenases/reductases (SDRs) and are translated as the two-domain flavoprotein oxygenase fusion with a separate C-terminal SDR [35, 43, 44]. LanM2 was shown to catalyze a thioesterase-like decarboxylative 2,3-dehydration of the ACP-tethered nascent angucycline generated by “minimal” PKS [44]. This reaction yields prejadomycin, which is recognized by further enzymes involved in the landomycins biosynthesis. The same reaction was proven in the *in vitro* studies for JadF during one-pot defucogilvocarcin enzymatic biosynthesis, where its removal led to accumulation of rabelomycin [45]. Thus, accumulation of rabelomycin by recombinant *S*. *albus* harboring pGRE may be due to the lack of GreM2 N-terminal oxidative activity. On the other hand, conversion of UWM6, the first intermediate in the angucycline’s biosynthesis, into rabelomycin was observed after incubation with LanE [44]. LanE homolog in grecocycline biosynthetic gene cluster is represented by GreE (74% identity with LanE and 76% identity with PgaE). Consequently, rabelomycin may originate from UWM6, which is recognized as a substrate by GreE. Only the oxygenase domain of GreM2 is required to release angucyclic intermediate tethered to ACP. One amino acid substitution (A2T) was identified in the sequence of *greM2* encoding N-terminal domain. This mutation occurred in the first amino acid after methionine and may not change enzymatic activity and folding of the GreM2 oxygenase domain. However, three amino acid substitutions were identified in the reductase domain encoding sequence of *greM2*, which would likely impair its catalytic activity. Therefore, we presume that mutated GreM2 may keep oxygenase activity and perform 2,3-dehydration of the ACP-tethered angucycline to give prejadomycin, which is recognized by GreE and (a) converted to dehydrorabelomycin or (b) in cooperation with GreV, generate tetrangulol.

Complementation of mutated *greM2* gene with the native gene copy had no impact on the production profile of *S*. *albus*. As was shown before, the balanced expression of genes encoding enzymes from the biosynthetic pathways is crucial for production levels [46, 47]. In this study we used a high-copy number plasmid and a strong synthetic promoter to enforce *greM2* transcription, while the whole *gre* cluster was present only in one copy per genome under the control of native promoters. Imbalance between copy-number and strength of promoters could explain why complementation did not trigger grecocycline production. Secondly, another three genes (*greD*, *greL* and *greGT4*) encoding enzymes involved in further biosynthetic steps had been mutated. Co-expression of these genes together with *greM2*, perhaps under the different conditions, might be needed for production of the glycosylated compounds. Another, and perhaps more plausible explanation, is that GreM2 actually functions at later step of the grecocycline biosynthesis, and cannot utilize precursors accumulating due to the lack of modifications by any or all GreD, GreL and GreGT4 enzymes.

Other nucleotide substitutions were located in the genes encoding GreA5, GreN, GreK, GreGT1, GreGT4, GreD, GreL and GreEx3 (Table 2). On two occasions mutations did not influence amino acid sequences (in GreGT1 and GreK). Clearly, the mutation in *greA5* did not have impact on the protein function, otherwise aromatic polyketides would not be formed. Most probably, mutation in GreN (I353V) also did not affect the enzyme activity significantly. GreN is highly similar (~80%) to different acetyl-CoA carboxyltransferases, and orthologs of this enzyme have been identified in many gene clusters of secondary metabolites [20, 48, 49]. For example, acyl-CoA carboxylase JadJ, from the jadomycin biosynthetic pathway, is supplying malonyl-CoA for the polyketide biosynthesis, and its inactivation severely reduced jadomycin B production [48]. Isoleucine and valine are highly similar amino acids and their swapping may not influence folding and activity of the enzyme. This is supported by significant yields of angucyclines observed after heterologous expression of re-assembled *gre* cluster. Therefore, it was concluded that mutation in *greN* is silent. As all other mutated enzymes/proteins are acting on the later stages of biosynthesis, it is impossible to predict whether they had any impact on the protein function. Nevertheless, it would explain why co-expression of the native *greM2* could not force grecocycline biosynthesis. Additionally, no mutations have been identified in any of the two regulatory genes, thereby GreR1 and GreR2 probably do not impair heterologous expression of pGRE.

In summary, TAR method is an efficient and rapid approach for assembly and re-assembly of large DNA fragments into entire biosynthetic gene cluster and its heterologous expression, although the PCR derived mutations should be taken into account. All experimental evidence suggests that the re-assembled gene cluster is indeed involved in the biosynthesis of grecocycline in the native producer and several unique enzymes (especially GreGT4 and the sugar biosynthetic proteins) may find their application in the derivatization of known active angucyclines via combinatorial biosynthesis.

## Supporting Information

S1 Fig Comparison of isolated tetrangulol and a standard. A—High-resolution mass spectra of the tetrangulol standard, B—UV/Vis spectrum of the tetrangulol standard, C—High-resolution mass spectra of an isolated tetrangulol, D—UV/Vis spectrum of an isolated tetrangulol.
(TIF)

S2 Fig Comparison of isolated rabelomycin and a standard. A—High-resolution mass spectra of the rabelomycin standard, B—UV/Vis spectrum of the rabelomycin standard, C—High-resolution mass spectra of an isolated rabelomycin, D—UV/Vis spectrum of an isolated rabelomycin.
(TIF)

S1 Table Oligonucleotides used in this study. (PDF)

S2 Table Oligonucleotides used for sequencing pGRE. (PDF)

S3 Table PCR conditions used in this study. (PDF)
