# Supplementary figures and images for: Cloning and Heterologous Expression of the Grecocycline Biosynthetic Gene Cluster
Source: PLoS One. 2016 Jul 13;11(7):e0158682. doi: 10.1371/journal.pone.0158682 (PMC4943663; doi:10.1371/journal.pone.0158682)

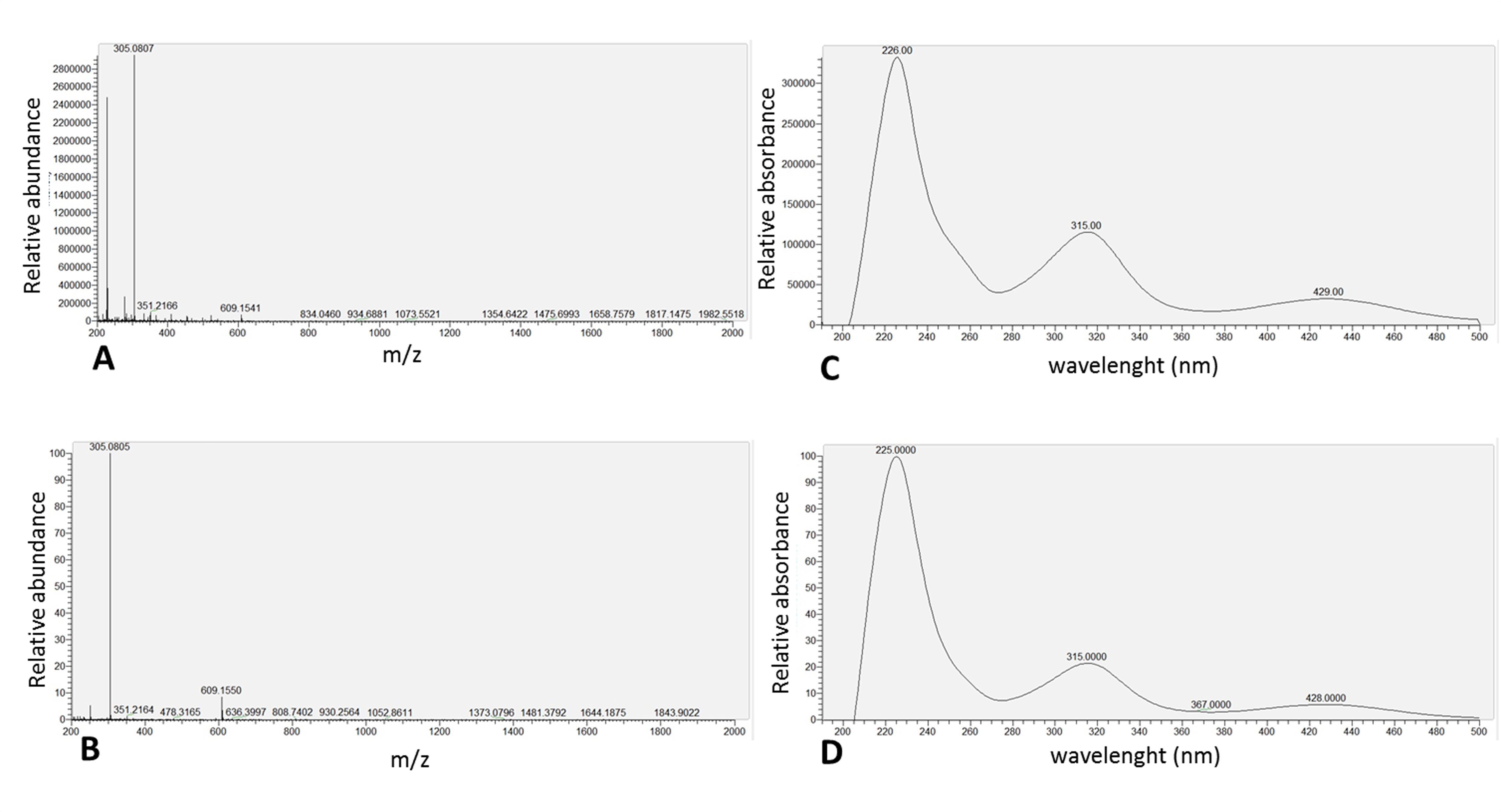

Supplement: S1 Fig — A—High-resolution mass spectra of the tetrangulol standard, B—UV/Vis spectrum of the tetrangulol standard, C—High-resolution mass spectra of an isolated tetrangulol, D—UV/Vis spectrum of an isolated tetrangulol. (TIF) [file pone.0158682.s001.tif]

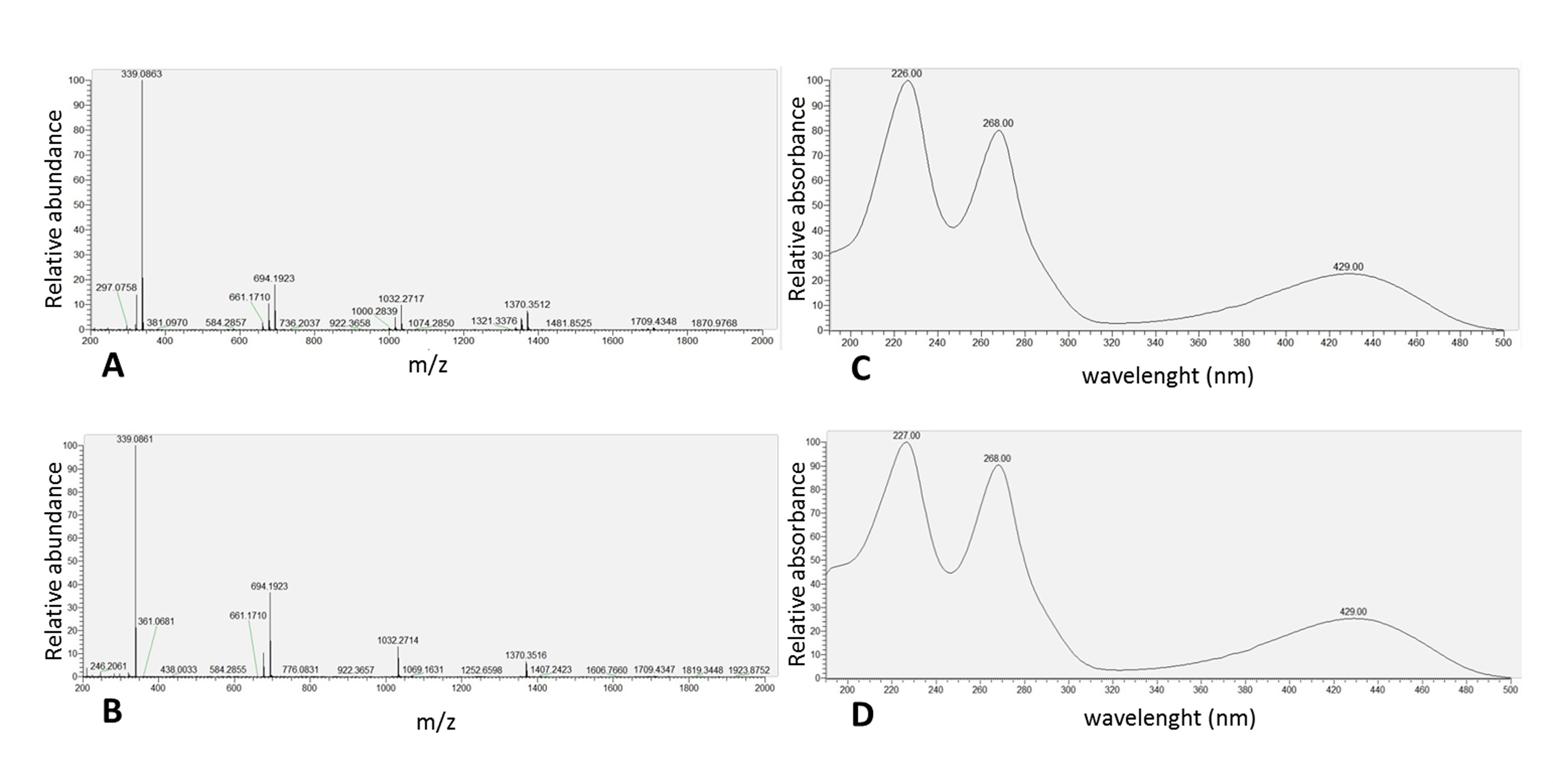

Supplement: S2 Fig — A—High-resolution mass spectra of the rabelomycin standard, B—UV/Vis spectrum of the rabelomycin standard, C—High-resolution mass spectra of an isolated rabelomycin, D—UV/Vis spectrum of an isolated rabelomycin. (TIF) [file pone.0158682.s002.tif]
