# Supplementary material for: Cloning and Heterologous Expression of the Grecocycline Biosynthetic Gene Cluster
Source: PLoS One. 2016 Jul 13;11(7):e0158682. doi: 10.1371/journal.pone.0158682 (PMC4943663; doi:10.1371/journal.pone.0158682)
Supplement: S1 Table — (PDF) [file pone.0158682.s003.pdf]

S1 Table. Oligonucleotides used in this study

Primer	Sequence (5'→ 3')	Restriction site
15aSOKf	CACAACATACGAGCCGGAAGCTCTACTAGAGTCAC ACTGGCTCACCTTCGGG	-
15aSOKr	GATGGAGCTGCACATGAACCGGGCTGACTTCAGGTG CTACATTTGAAGAGATAAATTGCACTG	-
SOK15af	GGTTCATGTGCAGCTCCATCAGC	-
SOK15ar	GCTTCCGGCTCGTATGTTGTGTGG	-
CEN_LEUf	GCCTCCGACTAACCCACCTGGGTCCTTTTCATCACGT GCTAT	-
CEN_LEUr	CGAAGAGCGATTGAGCTGATAGACGGTTTTTCGCCC TTGAC	-
CL9Lf	GGCGAAAAACCGTCTATCAGCTCAATCGCTCTTCGT TCGTCTGGAAGGC	-
CL9Lr	CGTGATGAAAAGGACCCAGGTGGGTTAGTCGGAGG CCAAACGGC	-
CLYbacf	TATTGACATGTCGTCGATTTCAGTGCAATTTATCTCT TCAAATG	-
CLYbacR	GAAATAAGATCACTACCGCACTTGGTGCACGCAGA G	-
BACclyf	GCGTGCACCAAGTGCGGTAGTGATCTTATTTCATTA TGGTGAAAGTTGG	-
BACclyr	GATAAATTGCACTGAAATCGACGACATGTCAATACT TGCCC	-
greTH-fF-SspI-f	TTT**AATATT**CGTGCACGGGCGGACGGTGTT[a]	*Ssp*I
greTH-fR-MCS-r	TTT**AATATT**AAGCCGCCGCCGCCGAGGACG[a]	*Ssp*I
greTH-fF-fus-r	CGGCGGGGGAAAGGCTTATG**GATATC**TTCCGAGCC TCCGTTGGGTG[a]	*Eco*RV
greTH-fR-fus-f	CACCCAACGGAGGCTCGGAA**GATATC**CATAAGCCT TTCCCCCGCCG[a]	*Eco*RV
greGT1-fR-f	CAGCACCGTACGGAGGCCTGA**GATATC**GCGGCACG	*Eco*RV

(fuz)	CCCGGACGGCCGG[a]	
greGT1-fF-f	CCC**AATATT**CCGGCCCGTTCCCCCTGACCG[a]	*Ssp*I
greGT1-fF-r (fuz)	CCGGCCGTCCGGGCGTGCCGC**GATATC**TCAGGCCTC CGTACGGTGCTG[a]	*Eco*RV
greGT1-fR-r	CCC**AATATT**GATCCGGAAGGCTCCAGGTCC[a]	*Ssp*I
pCLY10-Gre-st	TGGGCTGCAGGTCGACTCTAGAGGATCCGCGGCCGC GCCCCCCGGATGGCGCGGCTGTGTGGTGCTTCAAAG TGGC	-
pCLY10-Gre-end	TCGCGCCCTCCATGAGGCGTACCCGAAGTTCACCGA AGGTCGGACGCCGGGCCCCGGCCCGTCGATGCGCG GCGGG	-
greR1-f	GTGTGGTGCTTCAAAGTGGC	-
greR1-r	GCCCCGGGCGGTGCTCACTG	-
greR3-f	AGGGACCACCCCGTGAAAGC	-
greR3-r	AAGCTTTATGTGCTCATGCC	-
greR2.1-f	GGAGACCCGCCGGGTCCTGAT	-
greR2.1-r	GGAACACGTTGATCATTTCGT	-
greR2.2-f	CGGACTCCCACACGAGAGGAA	-
greR2.2-r	GCCCTTCTCCACACGGATGGT	-
greR1-R2.1-f	GTGGCCAACAACCCCGGCGCC	-
greR1-R2.1-r	TAGAGGGTCAGCAGCGCCACC	-
greR2.1.-2.2-f	CGGCTCGCCGAAGGAGCCCGA	-
greR2.1-2.2-r	GGTGCCTTCGACGGTGGAGGC	-
greR2.2-R3-f	ACTGGGCGGCGGAGCCCTGTG	-
greR2.2-R3-r	GGCGCCGGTGCGTTCCCGCAG	-

‘[a] – restriction sites introduced artificially are marked in bold
