# Supplementary material for: Cloning and Heterologous Expression of the Grecocycline Biosynthetic Gene Cluster
Source: PLoS One. 2016 Jul 13;11(7):e0158682. doi: 10.1371/journal.pone.0158682 (PMC4943663; doi:10.1371/journal.pone.0158682)
Supplement: S2 Table — (PDF) [file pone.0158682.s004.pdf]

S2 Table. Oligonucleotides used for sequencing pGRE

Primer	Sequence (5’→ 3’)
gre-seq-51.2	AGACGGTCACGGACAACCTGC
gre-seq-50.2	ATTCTGCTGGTCGTCTTCGGT
gre-seq-49.2	TAACTCATGGGTAAGCCCTTA
gre-seq-46.2	GGTGTACGTCGAAGGTGTTGA
gre-seq-43.2	TATGTGCAGATGCAGCAGGTC
gre-seq-41.2	AACCGTGGATGTACGAGAAGG
gre-seq-39.2	AGCTGTACTTGGCCGACATCC
gre-seq-36.2	AACTCTGTTCACTGCAAGCCG
gre-seq-35.2	TCAACTCCCTCACCCAGCACG
gre-seq-34.2	AACTACACCAAGGTCCACAAA
gre-seq-33.2	AACTCACCAACAGCGACATCA
gre-seq-27.2	GGGTGATCGGCAACGACATCA
gre-seq-24.2	AAGAACGCCATCGACTGGTTC
gre-seq-23.2	AGAACTCCAAGGTCAGCAGCA
gre-seq-18.2	GCTCATCAGCAGGTTCCTCTT
gre-seq-12.2	AACATCACCATCAACAGCGTG
gre-seq-7.1	TCATCGACACGAACCTCAACA
gre-seq-4.1	TGGCCATGAAGCACCACGTGG
gre-seq-1	ATCCGGTTGGTGTGTGATGTT
gre-seq-2	ATAGCTTGCCAGGAGCAGTTC
gre-seq-3	CGACGATGGGGCTCGACCAGG
gre-seq-4	GGTCGGGCACTCTCTGGGCGC
gre-seq-5	CCAGCGGAGTGGTCGTCAGCG
gre-seq-6	CCGCGGCCACGGCGGCTTCAA
gre-seq-7	CTGGTCAACAATGCGGGCCGC
gre-seq-8	GTGAGAAGGACGAACGCATCC
gre-seq-9	GCCGGTGTCGCCGAGGCCAGG
gre-seq-10	CCGGCGCTTCGAACGGCTGGA
gre-seq-11	GGCCTGCTGCTGAACATCTCC
gre-seq-12	TCTTCATCATCCAGAGGGCGC
gre-seq-13	GCTTCGGCGGACTGCTGCTGT

gre-seq-14	GATCCGCTCGGCGCTGGGGGC
gre-seq-15	GGAACGACGGCGGGCCGCTTC
gre-seq-16	CGCCACCGAGATCCAGCACCA
gre-seq-17	GACCGCTCGGACGACCCCGCC
gre-seq-18	TCCTGATCCGCTCCCTGGCCAT
gre-seq-19	GCACGGGCACCCGGCGGCACC
gre-seq-20	GGCGGGATGCGAGCGGGCTAT
gre-seq-21	CATTCCTGGAGGAGTGCCTCA
gre-seq-22	CAGGCGCGGCTGCGAGGTCCT
gre-seq-23	CCCCTGCGGGACGTGCTGTCC
gre-seq-24	ACGCCGATCCGGCGACGGAGG
gre-seq-25	GAGATGGAGGACGCCAACGCC
gre-seq-26	CGCCCCGGCGCGACCCGGACG
gre-seq-27	ACGCCGAACTCGCCGCGAGGG
gre-seq-28	CCCGGCAGATCAACTACTCCG
gre-seq-29	TCACCTACATCCCCCAGGACA
gre-seq-30	CGTCCTGGACGGTGCCATGAT
gre-seq-31	CCGAGAAGCTGATCCCGCTCT
gre-seq-32	GGCTCCTCGGCCAACCTGCTC
gre-seq-33	GGCTGCGGGAGGGACTGGACG
gre-seq-34	GCCAAGGAGTTCGGCGGCGTC
gre-seq-35	TCCTCGACACCGTGGAGCTGG
gre-seq-36	CTCATGGAGAACGTGATGTTC
gre-seq-37	CGGCGCTCGCCGCTGTCCTGC
gre-seq-38	GCTGCACTACGCCAAGTACCT
gre-seq-39	GCCACCGGCCCCACCGCGCCC
gre-seq-40	CGGCATGGTCTCCGGCCTCGA
gre-seq-41	GCTGGCGCCCGGACGTCGTGG
gre-seq-42	CCAGGAACTCCTGTCGAATCC
gre-seq-43	GCCCCGGTGCTCGCGAAGTTC
gre-seq-44	CGGAGGCCTGATGCGCGTCCT
gre-seq-45	ACCCTCGCCCTCAACGGCGTA
gre-seq-46	CCCGGAGACGGCCCGCCGTCC
gre-seq-47	ACGCCTTCACGGCGGACTGCC

gre-seq-48	CCGTGGCCGACGGGCCTCCTG
gre-seq-49	AGTCTGTCGGGCTGTGCAGCC
gre-seq-50	AAGGAGTTCGCCAAGGAGGGC
gre-seq-51	GCCGGCTGCCGGCGGAGGCGG
