# Supplementary material for: Cloning and Heterologous Expression of the Grecocycline Biosynthetic Gene Cluster
Source: PLoS One. 2016 Jul 13;11(7):e0158682. doi: 10.1371/journal.pone.0158682 (PMC4943663; doi:10.1371/journal.pone.0158682)
Supplement: S3 Table — (PDF) [file pone.0158682.s005.pdf]

S3 Table. PCR conditions used in this study

T °C	Time, min	
**PCR conditions for R1 and R3 amlification**		
95.0	03:00	
94.0	00:30	25
62.0	00:20	
68.0	13:00	
68.0	10:00	
4.0	-	
**PCR conditions for R2.1 and R2.2 amlification**		
98.0	03:00	
98.0	00:30	25
61.5	00:20	
72.0	05:00	
72.0	10:00	
4.0	-	
**PCR conditions for pGRE assembly verification**		
95.0	02:00	
95.0	00:17	25
57.0	00:15	
72.0	01:10	
72.0	05:00	
4.0	-	
